# Biometric and ICL-related risk factors associated with suboptimal vault with implantable collamer lenses

**DOI:** 10.3389/fmed.2026.1843957

**Published:** 2026-08-11

**Authors:** Xi Yang, Shan Yang, Zhikun Yang, Ying Li

**Affiliations:** 1 Department of Ophthalmology, The Affiliated Yongchuan Hospital of Chongqing Medical University, Chongqing, China; 2 Department of Ophthalmology, Peking Union Medical College Hospital, Chinese Academy of Medical Sciences and Peking Union Medical College, Beijing, China

**Keywords:** anterior chamber depth, biometry, crystalline lens rise, implantable collamer lens, vault

## Abstract

**Background:**

To comprehensively explore the biometry related to suboptimal vault (including low and high vault) after implantable collamer lens (ICL) implantation and the risk factors associated with ICL.

**Methods:**

This retrospective case-control study enrolled 180 patients with myopia and astigmatism who underwent EVO-V4c ICL implantation at Peking Union Medical College Hospital between January 2023 and December 2024. The normal vault group (NVG) was randomly selected from eligible patients during the same period to ensure balanced group sizes and reduce selection bias. Patients were divided into three groups based on the postoperative vault: low vault group (LVG, <250 μm, *n* = 36), high vault group (HVG, >750 μm, *n* = 72), and normal vault group (NVG, 250–750 μm, *n* = 72). Biometric parameters were collected before and after surgery for all patients, including anterior chamber depth (ACD), white-to-white (WTW), angle-to-angle (ATA), crystalline lens rise (CLR), axial length (AL), and spherical equivalent (SE). Multivariate analysis of variance (MANOVA), Pearson correlation analysis, multiple linear regression, and logistic regression analysis were employed to assess the relationship between each parameter and postoperative vault outcomes.

**Results:**

The results indicated significant differences in age, ACD, WTW, ATA, AL, and CLR among the three groups of patients (*P* < 0.05). Multivariate linear regression analysis revealed that ACD (*P* < 0.05), CLR (*P* < 0.001), and ATA (*P* = 0.018) were independent factors affecting postoperative vault. Logistic regression analysis showed that higher CLR (OR = 1.008, *P* = 0.013) was an independent risk factor for low vault, while deeper ACD (OR = 1.477, *P* = 0.030) and more negative ICL spherical equivalent (ICLSE, OR = 0.523, *P* = 0.034) were independent risk factors for high vault.

**Conclusions:**

Low vault is independently associated with greater CLR, whereas high vault is independently linked to deeper ACD and higher SE. Age, WTW, ATA, and AL also demonstrate significant but varying associations with vault type. These findings underscore the importance of integrating CLR and ATA measurements into routine preoperative assessment. Employing a more individualized sizing strategy—particularly in patients with deeper ACD or pronounced CLR—could enhance vault predictability and improve postoperative safety.

## Background

ICL have been widely used for the correction of myopia and myopic astigmatism, its efficiency, safety, and predictability have been proven by various studies (1–3). Vault, which refers to the vertical distance between the posterior surface of the ICL and the anterior surface of the natural crystalline lens, is one of the key parameters for evaluating the safety and long-term effectiveness of the surgery.

Shallow or excessive vaults are not complications in and of themselves but may increase the risk of complications. Low vault may increase the risk of contact between the ICL and the natural lens, thereby inducing subcapsular cataracts (4). Conversely, high vault may cause excessive dilation of the iris and narrowing of the anterior chamber angle, leading to pupillary block, pigment dispersion, secondary glaucoma, halos perception, and subjective optical abnormalities or discomfort in patients (5, 6). Therefore, achieving and maintaining an ideal and stable central vault is the core goal of successful ICL surgery.

Various studies have sought to predict the vault of an ICL using preoperative parameters (7, 8), yet suboptimal postoperative vaults persist in clinical practice. This study aims to comprehensively investigate the biometry and ICL-related risk factors associated with suboptimal vault and provide selective information regarding the anatomical and ICL features related to low and high vaults, thereby assisting surgeons in the thorough analysis of preoperative parameters.

## Methods

### Study design

This retrospective study enrolled patients implanted with an ICL for the correction of myopia and astigmatism (EVO-V4c, STAAR Surgical Co. Nidau, Switzerland) at Peking Union Medical College Hospital between January 2023 and December 2024. A total of 180 eyes of 180 patients were analyzed, of which 36 eyes of 36 patients with postoperative vault less than 250 μm were selected as the low vault group (LVG), and 72 eyes of 72 patients with postoperative vault between 250 and 750 μm were randomly selected as the normal vault group (NVG) from the database within the same observational period. Random selection was performed using a computer-generated random number sequence to ensure representativeness and minimize selection bias. To assess the robustness of our findings, we conducted a sensitivity analysis by repeating the logistic regression using all eligible normal vault cases (*n* = 156) instead of the randomly selected subset; the direction and significance of all identified risk factors remained consistent.

The inclusion criteria for the analysis were, spectacle refraction between −4.00 and −20.00 diopter sphere (DS), refractive astigmatism lower than −6.00 diopter cylinder (DC), internal anterior chamber depth (ACD: distance from corneal endothelium to crystalline lens anterior surface) >2.8 mm, endothelial cell count >2,000 cells/mm^2^ and the implanted ICL size was determined strictly based on the manufacturer's recommendations. The exclusion criteria were the presence of corneal abnormality such as ectasia, dystrophy or trauma, previous corneal refractive surgery and the presence of iris cysts. The study followed the tenets of the Declaration of Helsinki and approved by the Institutional Ethics Committee of PUMCH. All patients signed written informed consents before being enrolled in the current study.

### Preoperative and postoperative protocol

All patients underwent thorough preoperative and postoperative examinations, including uncorrected distance visual acuity (UDVA), corrected distance visual acuity (CDVA), subjective refraction, non-contact intraocular pressure, slit-lamp microscopy and funduscopic examination. Anterior segment optical coherence tomography (AS-OCT; Visante, Zeiss Meditec AG, Jena, Germany) was used for measuring the horizontal anterior-chamber angle-to-angle distance (ATA), crystalline lens rise (CLR), ACD and postoperative vault. AS-OCT was performed under undilated natural-pupil conditions in a standardized dim-light environment of approximately 5 lux by an experienced technician. All AS-OCT images were manually reviewed and measured by the same technician. Scheimpflug photography (Pentacam HR, OCULUS Optikgeräte GmbH, Wetzlar, Germany) was used for quantifying the central keratometry (Kc) and the central corneal thickness (CCT). IOLMaster 700 (Carl Zeiss Meditec AG, Jena, Germany) was used for measuring axial length (AL) and horizontal white to white (WTW). Endothelial cell count was performed with a noncontact specular microscopy (Topcon SP-2000P, Topcon Corporation, Tokyo, Japan) using a 12-point sampling. The ICL size and dioptric power were determined using the manufacturer's calculator.

Patients were followed up on postoperative 1 day, 1 week, and 1, 3 months. UDVA, intraocular pressure measurements, and slit-lamp examination for subjective vault and any adverse effects were obtained on each visit. Central vault was measured using AS-OCT at 1 and 3 months postoperatively. Given that previous studies have demonstrated that vault stabilizes by 3 months after surgery (9, 10), the 3-month vault measurements were used as the primary data for the final statistical analysis.

### Surgical protocol

The surgery was performed under local anesthesia using 2% intracameral Lidocaine (B.Braun^®^ 20 mg/ml). The anterior chamber was filled with viscoelastic and the ICL introduced through a 3.2 mm temporal clear corneal incision using the manufacturer's injector cartridge. Upon positioning the ICL, the viscoelastic was removed through aspiration from the anterior chamber. After surgery, antibiotic (Exocin^®^ Ofloxacin 3 mg/ml), corticoid (Predforte^®^ Prednisolone acetate 10 mg/ml) and anti-inflammatory (Voltaren^®^ Diclofenac sodium 1 mg/ml) drugs were prescribed four times a day for 3 weeks.

### Statistical analysis

Statistical analyses were performed with SPSS software version 25.0 (SPSS, Inc). Differences among groups were investigated using MANOVA and pairwise comparisons were accounted for by applying the Bonferroni correction. Prior to multiple linear regression, we performed univariate linear regression to screen candidate variables (*P* < 0.10). Variables with pairwise correlation coefficients >0.6 were examined for multicollinearity; the variance inflation factor (VIF) for all included variables was below 2.5, indicating no significant multicollinearity. The relationship between the vault values and biometric parameters was evaluated using multiple linear regression analysis. Conditional logistic regression analysis was applied to identify the variables relevant for characterizing the type of vault, with model fit assessed by the Hosmer-Lemeshow test (*P* = 0.412) and Nagelkerke *R*^2^ = 0.387. The NVG was chosen as the reference group. A *P* value of less than 0.05 was considered statistically significant.

## Results

The demographic and clinical baseline characteristics of the study participants for the entire sample and for the three groups are summarized in Table 1. Group comparison showed statistically significant differences among the three vault groups in age, ACD, WTW, ATA, AL, CLR and vault (all *P* < 0.05). There were no significant differences in gender distribution, central keratometry, CCT, or SE among the three groups.

**Table 1:** Comparison of demographics and ocular characteristics of patients among different vault groups.

Parameter	All	LVG	NVG	HVG	*P* value
*N*	180	36	72	72	-
Age (years)	28.5 ± 5.7	31.7 ± 6.4	28.4 ± 5.7	27.0 ± 4.8	<0.01
Gender (M/F)	24/156	6/30	12/60	6/66	0.27
SE (D)	−9.09 ± 2.92	−8.85 ± 3.20	−8.89 ± 2.65	−9.41 ± 3.03	0.483
CCT (μm)	520.1 ± 32.9	516.5 ± 32.9	520.5 ± 32.1	521.5 ± 34.1	0.758
Kmean (D)	43.91 ± 1.40	44.44 ± 1.58	43.78 ± 1.23	43.77 ± 1.41	0.05
ACD (mm)	3.20 ± 0.27	3.02 ± 0.23	3.18 ± 0.23	3.32 ± 0.27	<0.01
WTW (mm)	12.00 ± 0.47	11.82 ± 0.51	11.99 ± 0.42	12.09 ± 0.49	0.019
ATA (mm)	11.72 ± 0.48	11.48 ± 0.58	11.71 ± 0.43	11.83 ± 0.44	0.01
CLR (μm)	−22.7 ± 190.8	136.3 ± 185.3	−27.3 ± 160.8	−97.6 ± 174.5	<0.01
AL (mm)	27.0 ± 1.5	26.3 ± 1.5	27.0 ± 1.3	27.3 ± 1.7	0.006
ECD (cell/mm^2^)	2,751.3 ± 259.5	2,792.7 ± 292.9	2,728.1 ± 244.8	2,753.9 ± 257.2	0.475
Vault (mm)	0.62 ± 0.28	0.20 ± 0.04	0.57 ± 0.12	0.88 ± 0.12	<0.01
ICLSE (D)	−10.67 ± 2.85	−10.33 ± 3.07	−10.54 ± 2.56	−10.97 ± 3.02	0.494
ICL size-WTW (mm)	0.84 ± 0.41	0.96 ± 0.48	0.82 ± 0.35	0.80 ± 0.42	0.134
ICL size-ATA (mm)	1.13 ± 0.34	1.31 ± 0.37	1.10 ± 0.29	1.06 ± 0.33	<0.01
ICL size (mm)					0.11
12.1	6	3	3	0	
12.6	101	21	42	38	
13.2	68	10	26	32	
13.7	5	2	1	2	

Values are expressed as mean ± SD or *n* (%). LVG, low vault group; NVG, normal vault group; HVG, high vault group; SE, spherical equivalent; CCT, central corneal thickness; Kmean: mean keratometry; ACD, anterior chamber depth; WTW, white-to-white; ATA, angle-to-angle; CLR, crystalline lens rise; AL, axial length; ECD, endothelial cell count; ICLSE, ICL spherical equivalent.

*P* values were derived from ANOVA or χ^2^ test as appropriate.

**Figure 1:**
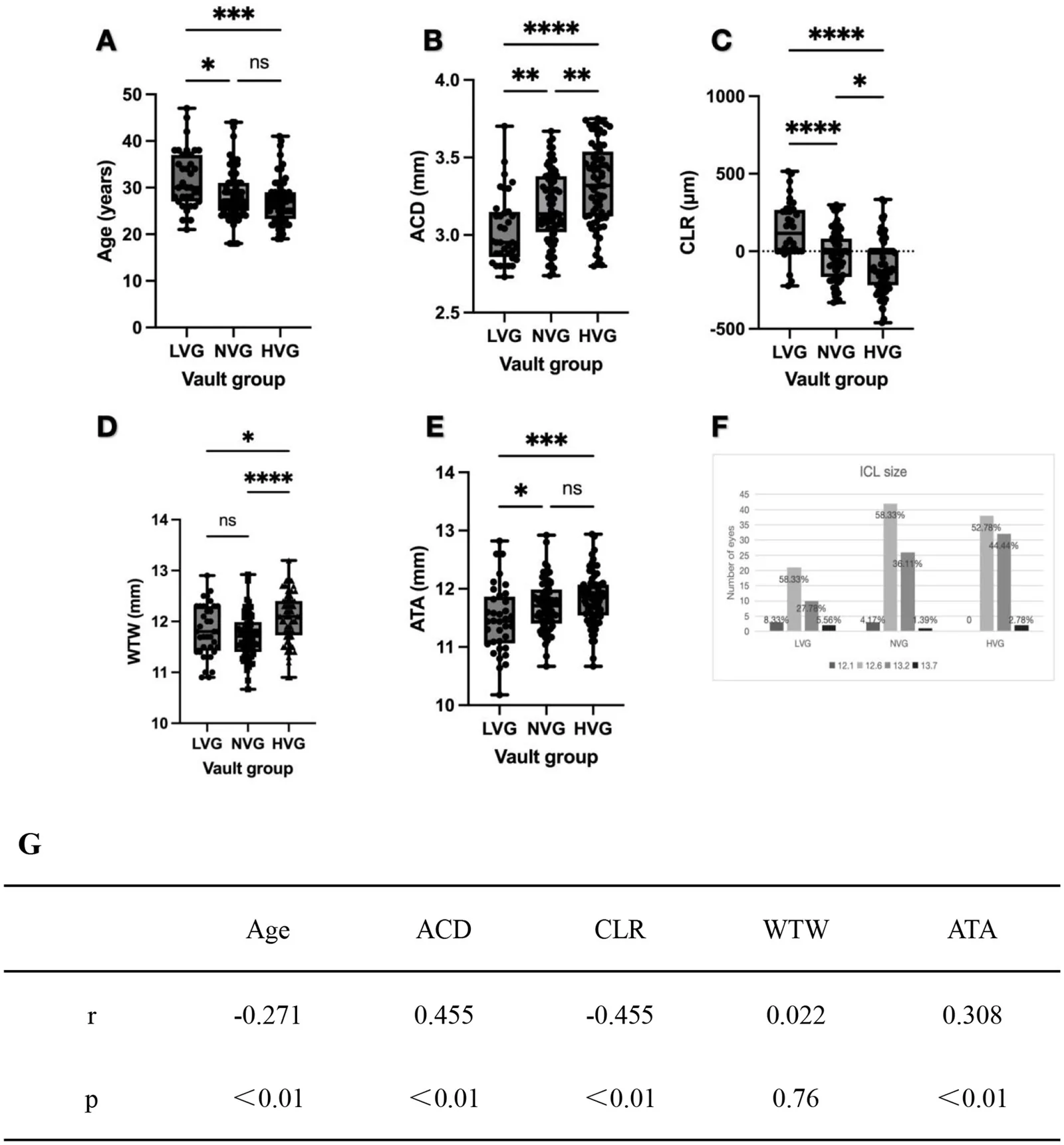
Pairwise comparisons between different groups. **A**: Patient age in years; **B**: Internal anterior chamber depth (ACD) in millimeters; **C**: Crystalline lens rise (CLR) in micrometers; **D**: white to white (WTW) in millimeters; E: angle to angle (ATA) in millimeters; **F**: Number of eyes in each vault group per ICL size. In figure F the percentages indicate the proportion of eyes implanted with ICL 12.1, 12.6, 13.2 and 13.7 mm in the three vault groups. Pearson correlation of vault and different parameters; **G**: Pearson correlation analysis between vault and preoperative parameters.

For variables showing statistically significant group differences, pairwise comparisons were supplemented by correlation analyses. Age was negatively correlated with vault (Pearson's *r* = −0.271, *P* < 0.001; Figure 1A), whereas ACD was positively correlated with vault (*r* = 0.455, *P* < 0.001; Figure 1B). With regard to the CLR, eyes in the LVG had more protruded crystalline lenses compared to the eyes in the NVG and HVG (Figure 1C). WTW, and ATA were significantly lower in the LVG compared to the remaining two groups (Figures 1D, E). There were no significant differences in the proportion of ICL size, among the three groups (Figure 1F).

All variables were strictly selected before being included in the regression model using simple linear regression. If the correlation coefficient of the two variables was greater than 0.6, only one variable was selected in the analysis; Pearson correlation analysis revealed that ATA was highly correlated with ICLsize—ATA (*r* = 0.693), and WTW with ICLsize—WTW (*r* = 0.700). To avoid multicollinearity, we excluded the derived mismatch indices and retained only ATA and WTW in the final model. Multiple linear regression analysis revealed that ACD (*P* < 0.05), CLR (*P* < 0.001), and ATA (*P* = 0.018) were the three explanatory variables associated with the postoperative vault (Table 2).

**Table 2:** Multiple linear regression analysis evaluating the association between preoperative biometric parameters and central vaulting after ICL implantation.

Parameters	Unstandardized coefficient	Standardized coefficient	*P*	95% CI
Age (years)	−0.005	−0.102	0.153	−0.012~0.002
CCT (μm)	0.000	0.039	0.561	−0.001~0.001
Kmean (D)	0.034	0.172	0.120	−0.009~0.077
ACD (mm)	0.178	0.175	0.047^*^	0.002~0.353
WTW (mm)	−0.029	−0.051	0.584	−0.135~0.077
ATA (mm)	0.129	0.225	0.018^*^	0.023~0.235
CLR (μm)	0.000	−0.320	<0.001^*^	−0.001~0.000
AL (mm)	0.042	0.233	0.117	−0.011~0.095
ICLSE (D)	0.008	0.087	0.473	−0.015~0.032

CCT, central corneal thickness; Kmean, mean keratometry; ACD, anterior chamber depth; WTW, white-to-white; ATA, angle-to-angle; CLR, crystalline lens rise; AL, axial length; ICLSE, ICL spherical equivalent. ACD, CLR, and ATA were identified as significant predictors of postoperative vault. *P* < 0.05 indicates statistical significance.

A stepwise multinomial logistic regression analysis (Table 3) was used to identify the predictive factors for the presence of sub-optimal vault after ICL implantation. The model used the optimal vault range as reference and identified the CLR, ACD, and ICLSE as relevant factors for the presence of a low or high vault. With respect to the risk of developing low vaults, this increased in eyes with a higher CLR (OR: 1.008, 95% CI: 1.002–1.015, *P* = 0.013). As far as the risk of high vault is concerned, eyes with deeper ACD (OR: 1.477, 95% CI: 1.035–2.108, *P* = 0.030) and more negative ICLSE (OR: 0.523, 95% CI: 0.287–0.952, *P* = 0.034) had a higher chance of presenting high vaults (Table 3). Notably, the OR for ACD in the high vault group was substantially larger than that for the low vault group, suggesting a stronger influence of anterior chamber depth on excessive vault.

**Table 3:** Multinomial logistic regression model summary.

Parameters	Low vault group (*N* = 36)				High vault group (*N* = 72)			
	β1	OR	*p*	OR 95% CI	β2	OR	*p*	OR 95% CI
Age (years)	0.121	1.129	0.09	0.981–1.298	−0.051	0.950	0.231	0.874–1.033
CCT (mm)	−0.007	0.993	0.544	0.970–1.016	−0.006	0.994	0.370	0.982–1.007
CLR (μm)	0.008	1.008	0.013^*^	1.002–1.015	−0.002	0.998	0.287	0.995–1.001
WTW (mm)	−0.286	0.751	0.905	0.007–82.936	0.148	1.159	0.885	0.156-−8.606
ATA (mm)	−0.770	0.463	0.501	0.049–4.362	0.618	1.856	0.451	0.372–9.252
Kmean (D)	−0.537	0.585	0.314	0.205–1.663	−0.402	0.669	0.230	0.347–1.290
AL (mm)	−1.322	0.267	0.076	0.062–1.147	−0.908	0.403	0.061	0.156–1.044
ACD (mm)	1.182	3.261	0.631	0.026–403.375	0.390	1.477	0.030^*^	1.035–2.108
ICLSE (D)	−0.344	0.709	0.231	0.404–1.245	−0.649	0.523	0.034^*^	0.287–0.952

^*^P < 0.05 indicates statistical significance. OR, odds ratio; CI, confidence interval; CCT, central corneal thickness; CLR, crystalline lens rise; WTW, white-to-white; ATA, angle-to-angle; Kmean, mean keratometry; AL, axial length; ACD, anterior chamber depth; ICLSE, ICL spherical equivalent.

## Discussion

Several parameters have been reported to be associated with postoperative vault after ICL implantation, including WTW, ACD, lens thickness, pupil size, ciliary body morphology, and ICL dioptric power (11–13). However, studies specifically investigating risk factors for suboptimal vault—particularly the extremes of low and high vault—remain limited. In this study, we systematically evaluated the biometric and ICL-related determinants of suboptimal vault following ICL implantation. Our findings reveal distinct risk profiles for low and high vaults. A more protruded crystalline lens (higher CLR) emerged as the primary independent risk factor for low vault, whereas a deeper ACD and more negative ICLSE were independently associated with an increased risk of high vault. These results underscore the multifactorial nature of postoperative vault and highlight the need for a more nuanced preoperative assessment that extends beyond traditional parameters.

Compared with the prior study by Cerpa Manito et al. (14), which also identified CLR and ACD as predictors of suboptimal vault, our study provides several novel contributions. First, we specifically quantified the odds ratios for low and high vault using a multinomial logistic model with a randomly selected normal vault group, thereby reducing selection bias and enabling direct comparison between both extremes of vault. Second, our analysis identified ATA—rather than WTW—as a significant independent predictor of vault in linear regression, reinforcing the superiority of internal anatomical landmarks for ICL sizing. Third, we clarified the direction of the association between ICLSE and high vault, showing that more negative ICLSE (i.e., higher myopia) increases risk, which has practical implications for preoperative planning in high myopes.

The strong association between CLR and low vault is a key finding with clear anatomical rationale. The crystalline lens rise quantifies the anterior projection of the lens relative to the angle-to-angle plane. A higher CLR indicates a more anteriorly positioned or thicker lens, which inherently reduces the space available posterior to the ICL, thereby increasing the likelihood of a low vault (14). This aligns with the work of Cerpa Manito et al. (14), who identified high CLR as a major risk factor for low vault. Our logistic regression model quantified this risk, showing that for every micrometer increase in CLR, the odds of a low vault increased by 0.8% (OR 1.008, *P* = 0.013). This suggests that in eyes with a pronounced CLR, even a standard-sized ICL may sit closer to the crystalline lens, potentially necessitating a different sizing strategy or a more cautious surgical approach.

In contrast, high vault was predominantly driven by a deeper ACD and more negative ICLSE. The role of ACD is intuitive: a deeper anterior chamber provides a larger overall space for the ICL to reside, allowing it to assume a more posterior position relative to the cornea and, consequently, a higher vault relative to the lens (15, 16). However, our finding that deeper ACD is a risk factor for high vault (OR 1.477, *P* = 0.030) also points to a potential limitation in current sizing algorithms. These algorithms, while generally safe, may tend to recommend larger ICLs for eyes with deeper ACDs to ensure adequate angle clearance. In some cases, this could lead to an overestimation of the required lens size, resulting in an excessively high vault. This highlights a clinical dilemma: the need to balance the risk of low vault (by choosing a larger lens) against the risk of high vault in eyes with already deep chambers. The association with more negative ICLSE (OR 0.523, *P* = 0.034) is noteworthy. Because ICLSE represents the implanted lens power, which is highly correlated with the degree of myopia, more negative values indicate higher myopic correction. In eyes with higher myopia, the ICL is often thicker or has a different curvature, potentially influencing its final position and vault. This finding suggests that surgeons should consider not only anatomical dimensions but also the lens power itself when anticipating vault outcomes.

The ongoing debate regarding the optimal parameter for ICL sizing is central to our findings. While WTW remains the manufacturer-recommended standard, its limitations are well-documented, with studies showing that up to 25% of cases sized by WTW alone result in vaults outside the optimal range (17). Our multiple linear regression analysis identified ATA, not WTW, as a significant independent predictor of vault (*P* = 0.018). This finding lends strong support to the growing body of evidence advocating for ATA as a superior surrogate for the true anatomical “sizing” landmark: the sulcus-to-sulcus (STS) diameter (18, 19). The ICL haptics are designed to rest in the ciliary sulcus, making STS the most directly relevant anatomical measurement. However, its clinical utility is hampered by the need for contact UBM and its variable repeatability (20). ATA, measured non-invasively and reproducibly with AS-OCT, provides a highly correlated and clinically practical proxy for STS. Our results suggest that incorporating ATA into the sizing decision-making process could significantly enhance vault predictability, moving beyond the corneal landmark of WTW to an internal angle-based measurement that more closely reflects the ICL's final resting place.

The observed inverse relationship between age and vault, with older patients more likely to be in the LVG, is consistent with previous longitudinal studies (10, 21). This is likely a manifestation of age-related physiological changes within the eye. As the crystalline lens continues to grow throughout life, it increases in both thickness and anterior curvature, leading to a progressive increase in CLR (22). Our data supports this mechanism, as LVG patients were not only older but also had significantly higher CLR values. Furthermore, age-related changes in iris dynamics and ciliary body position may also subtly alter the posterior chamber anatomy over time (23, 24). This finding reinforces that vault is not a static entity but one that exists within a dynamic, aging anatomical environment. While we cannot modify a patient's age, understanding this trend allows surgeons to better anticipate long-term vault stability, particularly in younger patients where a slightly higher early vault might be more acceptable given the expected age-related decline.

From a clinical application perspective, our findings support a more individualized ICL sizing strategy. For patients with a high CLR (>100 μm), even if WTW and ATA measurements suggest a standard lens size, surgeons may consider selecting a slightly larger ICL to mitigate the risk of low vault, particularly in older patients. Conversely, for patients with a deep ACD (>3.3 mm) and high myopia requiring a negative ICLSE beyond −12.0 D, caution should be exercised to avoid oversizing, as these eyes are at significantly higher risk of excessive vault. Incorporating ATA measured by AS-OCT into routine preoperative assessment, alongside CLR, could improve the accuracy of vault prediction and reduce the incidence of suboptimal outcomes.

The strengths of this study lie in its comprehensive evaluation of multiple biometric parameters and its focus on the extremes of vault, which are of greatest clinical concern. However, several limitations must be acknowledged. First, its retrospective design introduces potential selection bias. To mitigate this, we randomly selected the NVG and performed a sensitivity analysis confirming the robustness of our findings. Second, the follow-up duration was relatively short, and vault may undergo changes over the long term postoperatively; therefore, longer-term follow-up studies are needed to validate our conclusions. Third, our analysis was based on static, preoperative measurements. The final ICL vault is also influenced by dynamic factors such as accommodative status, pupil size, and lens thickness, which were not assessed. Future research should aim to develop predictive models that incorporate both static anatomical factors and dynamic variables. The development of such models, potentially leveraging machine learning, holds the key to achieving truly personalized ICL sizing and eliminating suboptimal vault outcomes.

## Conclusions

This study identifies distinct biometric predictors for suboptimal vault following ICL implantation. Low vault is independently associated with greater CLR, whereas high vault is independently linked to deeper ACD and more negative ICLSE. Age, WTW, ATA, and AL also showed significant but varying associations with vault type. These findings underscore the importance of integrating CLR and ATA measurements into routine preoperative assessment. Employing a more individualized sizing strategy—particularly in patients with deeper ACD or pronounced CLR—could enhance vault predictability and improve postoperative safety.

## Data Availability

The raw data supporting the conclusions of this article will be made available by the authors, without undue reservation.
